# Genomic analysis of circular RNAs in heart

**DOI:** 10.1186/s12920-020-00817-7

**Published:** 2020-11-07

**Authors:** Kunzhe Dong, Xiangqin He, Huabo Su, David J. R. Fulton, Jiliang Zhou

**Affiliations:** 1 Department of Pharmacology and Toxicology, Medical College of Georgia, Augusta University, 1459 Laney Walker Blvd, Augusta, GA 30912 USA; 2 Vascular Biology Center, Medical College of Georgia, Augusta University, Augusta, GA 30912 USA

**Keywords:** Circular RNA, Dilated cardiomyopathy, Read-through circRNA, miRNA sponge

## Abstract

**Background:**

Heart failure is a leading cause of human morbidity and mortality. Circular RNAs (circRNAs) are a newly discovered class of RNA that have been found to have important physiological and pathological roles. In the current study, we de novo analyzed existing whole transcriptome data from 5 normal and 5 dilated cardiomyopathy (DCM) human heart samples and compared the results with circRNAs that have been previously reported in human, mouse and rat hearts.

**Results:**

Our analysis identifies a list of cardiac circRNAs that are reliably detected in multiple studies. We have also defined the top 30 most abundant circRNAs in healthy human hearts which include some with previously unrecognized cardiac roles such as circHIPK3_11 and circTULP4_1. We further found that many circRNAs are dysregulated in DCM, particularly transcripts originating from DCM-related gene loci, such as *TTN* and *RYR2*. In addition, we predict the potential of cardiac circRNAs to sponge miRNAs that have reported roles in heart disease. We found that circALMS1_6 has the highest potential to bind miR-133, a microRNA that can regulate cardiac remodeling. Interestingly, we detected a novel class of circRNAs, referred to as read-though (rt)-circRNAs which are produced from exons of two different neighboring genes. Specifically, rt-circRNAs from *SCAF8* and *TIAM2* were observed to be dysregulated in DCM and these rt-circRNAs have the potential to sponge multiple heart disease-related miRNAs.

**Conclusions:**

In summary, this study provides a valuable resource for exploring the function of circRNAs in human heart disease and establishes a functional paradigm for identifying novel circRNAs in other tissues.

## Background

Heart failure (HF) is a leading cause of human morbidity and mortality worldwide. Despite advances in the management of HF, it remains a tremendous and growing public health burden in an aging population with an estimated prevalence of > 37.7 million individuals globally [1]. Dilated cardiomyopathy (DCM), characterized by the dilation and impaired contraction of the left or both ventricles, is a primary cause of HF and sudden cardiac death [2]. Understanding the etiology of DCM is vitally important in the search for better therapeutic approaches for HF.

In addition to non-genetic causes, such as hypertension, inflammation, and toxins, genetic factors are increasingly recognized for their roles in HF susceptibility [3, 4]. Currently, mutations in more than 100 genes have been linked to DCM [5]. However, these DCM relevant gene variants are only detected in ~ 37% of DCM patients [6]. Therefore, it is likely that other unknown genetic factors await discovery. The aberrant expression of different types of RNA transcripts, including those transcribed from protein coding genes [7–9], long non-coding genes [10, 11] and miRNAs [12–14], have been observed in DCM.

Circular RNAs (circRNAs) are a novel class of RNA that are generated by the covalent ligation of the 5′ and 3′ ends of a single-stranded RNA transcript to form a circular transcript [15]. CircRNAs are stable and resistant to RNase R, widespread throughout the plant and animal kingdoms and conserved across multiple species. Furthermore, circRNAs can be expressed in a tissue-specific manner [16, 17]. These properties make circRNAs excellent candidates as potential biomarkers for the diagnosis of disease and therapeutic intervention. Emerging evidence has demonstrated that circRNAs play important roles in various physiological and pathological processes, such as myogenesis [18], phenotypic modulation of smooth muscle cells [19, 20], atherosclerosis [21, 22], and cancer [23–25].

Although several efforts have been made to characterize the circRNAs expressed in the heart [26–29], our understanding of the importance of cardiac circRNAs is still very limited. Furthermore, several fundamental questions remain to be addressed. First, there is a large discrepancy between the presence and abundance of cardiac circRNAs that have been identified in previous studies [27–29], which limits any interpretation of their importance. To pinpoint truly important cardiac circRNAs, a cross-reference analysis between multiple independent studies is required. Second, it has been documented that circRNAs function primarily as molecular sponges to competitively bind miRNAs or encode small peptides. While the coding ability of cardiac circRNAs have been investigated recently by analyzing translational Ribo-seq data from human hearts [28], the potential of cardiac circRNAs to function as miRNA sponges remains incompletely understood. Finally, read through-circRNAs (rt-circRNAs), a recently discovered type of circRNAs that are produced from the exons of two adjacent genes on the same strand, have been implicated in both normal and tumor tissues [30], yet whether this type of rt-circRNAs are expressed in the human heart remains to be identified.

To address these questions, we de novo analyzed publicly available RNA-seq datasets from human hearts. These data sets were of sufficient sample size (5 normal controls and 5 DCM patients) [10], to enable us to investigate the general landscape of human cardiac circRNAs under homeostatic (normal) and disease settings. By integrating the analysis of circRNAs identified in human hearts with that of mouse and rat hearts in previous studies [26, 27, 29], we: (i) characterized the general features of circRNAs in human hearts and compared the identified circRNAs with circRNAs reported by previous studies and databases; (ii) determined the key circRNAs expressed in healthy hearts as defined by the top 30 most abundant circRNAs; (iii) identified circRNAs that are dysregulated in DCM hearts; (iv) predicted the potential of high-confidence cardiac circRNAs to act as a sponge for previously identified heart disease-related miRNAs; Lastly, we identified a list of rt-circRNAs, especially those from the *SCAF8* and *TIAM2* gene loci that are dysregulated in DCM and show strong potential of being miRNA sponges. Our work identifies a set of highly reliable circRNAs with potentially important roles in maintaining cardiac homeostasis which is a valuable resource for exploring the function of circRNAs in human heart disease. Our work further establishes a paradigm for identifying novel circRNAs in other tissues.

## Methods

### Identification of circRNAs

RNA-seq raw reads from the left ventricle of 5 DCM patients and 5 normal controls were obtained from SRA database (SRP108571) [10] and analyzed as illustrated in Additional file 1: Figure S1. Low-quality reads were first removed by Trimmomatic 3.0 [31]. The clean reads were then mapped to the GRCh38 human genome reference by Bowtie2 [32]. For the linear RNA analysis, the raw count of each gene was calculated by using HTSeq [33] and Fragments Per Kilobase of exon per Million fragments mapped (FPKM) values were calculated from raw counts to quantify expression of genes [34]. Differential analysis of linear RNA between DCM and normal samples was performed with DESeq2 [35] and genes with fold change (FC) > 2 and FDR < 0.05 were considered significance. Next, circRNAs were identified following the instruction of find_circ [36]. Briefly, by using SAMtools the unmapped reads were retrieved [37]. Then these unmapped reads were cut off 20 mers from both ends and mapped to the human genome independently in order to find unique anchor positions. Anchors that aligned in the reversed orientation suggest a back-spliced junction. CircRNAs with ≥ 2 unique back spliced reads in at least one sample were saved for analysis of circRNA features. The expression of circRNAs was normalized to Spliced Reads per Billion Mapped Reads (SRPBM). For identification of the differentially expressed circRNAs between normal and DCM samples, only those high confidence circRNAs, with ≥ 2 unique reads in at least four samples from at least one group were used. Differentially expressed circRNAs were identified with edgeR package based on a negative binomial distribution model [38]. CircRNAs with FC > 2 and *p* value < 0.05 were considered significant. Pathway analysis was performed using Metascape [39].

### Comparison with other studies

Results from three previous studies [26–29], which identified circRNAs in human, mouse or rat hearts, as well as four public circRNA databases, including circBase [40], circBank [41], circRNADB [42], CIRCpedia V2 [43], were used in comparison with our results. The R package liftOver (https://www.bioconductor.org/packages/release/workflows/html/liftOver.html) was used to convert start and end coordinates (hg19, mm9, mm10 and rn5) to hg38 first and then the converted coordinates were used for cross-reference and cross-species comparison. The venn diagrams were generated with online tool (https://bioinformatics.psb.ugent.be/webtools/Venn/).

### Analysis of circRNAs from two different genes

The 50-bp sequences from each side of the back-spliced junction of the cricRNAs arising from the exons of two different genes (gene 1 and gene 2), were retrieved and used as query sequences to perform BLAST analysis against human cDNA sequences downloaded from ENSEMBL database with default parameters. If any of the two sequences could be aligned to both gene 1 and gene 2, the circRNA was considered as possible false positive due to the sequence homologue of gene 1 and gene 2. If the sequence from each side of the back-spliced junction returned BLAST hits only matching its expected host gene, the circRNA was considered as true rt-circRNA.

### Construction of circRNA-miRNA-mRNA regulatory network

A list of 50 miRNAs which have been implicated in heart disease were obtained (References were provided in Additional file 2: Table S1). Seed sequence and potential targeted genes of these miRNAs was obtained from TargetScan database (https://www.targetscan.org/). CircRNA sequence was retrieved using custom R script and TargetScan was used for predicting the binding sites of the selected miRNAs. CircRNAs with at least 5 binding sites for at least one miRNA were considered to have potential to sponge miRNAs**.** The circRNA-miRNA-mRNA regulatory network was visualized using Cytoscape (https://cytoscape.org/).

### Experimental validation of circRNAs

Due to the lack of availability of human samples, the mouse orthologs of 6 identified human circRNAs were used for experimental validation. Mouse heart RNA was extracted with TRIzol reagent (Invitrogen) and reverse transcribed using random hexamers and High Capacity RNA-to-cDNA Kit (Invitrogen). Convergent primers with opposite orientation were designed (Additional file 3: Table S2) and used for PCR amplification on mouse heart cDNA and genomic DNA. Mouse *Yap1* gene primers were used as a positive control for PCR amplification of genomic DNA. PCR products were then subjected to Sanger sequencing.

## Results

### General features of circRNAs in both healthy and DCM human hearts

We performed a de novo analysis of public RNA-seq data sets that were generated from left ventricular tissues obtained from 5 healthy individuals and 5 DCM patients [10]. These data sets were selected because they were generated using a ribosome-depletion library, which is the gold standard method to identify back-spliced junctions for circRNAs detection [44]. We first removed low-quality reads and then utilized Bowtie2 [32] to align clean reads against the human reference genome. Reads continually aligned to the genome were used for linear gene expression analysis and the remaining un-mapped reads were further used as input for find_circ [36] to detect both linear and back-spliced junctions (Additional file 1: Figure S1). We first performed a quality control validation of this data by performing principal component analysis (PCA) based on the expression of linear genes with raw count > 10 in at least 5 samples. PCA analysis confirmed that samples from each group were clustered respectively as expected (Additional file 4: Figure S2A). We further examined the expression of a marker of HF, atrial natriuretic peptide α (*NPPA*). Consistent with the reported HF phenotype [45], our analysis revealed that the expression of *NPPA* is significantly increased in DCM specimens (Additional file 4: Figure S2B). Taken together, these initial analyses confirm the quality of this RNA-seq dataset and its suitability for the further analysis of circRNAs.

**Fig. 1:**
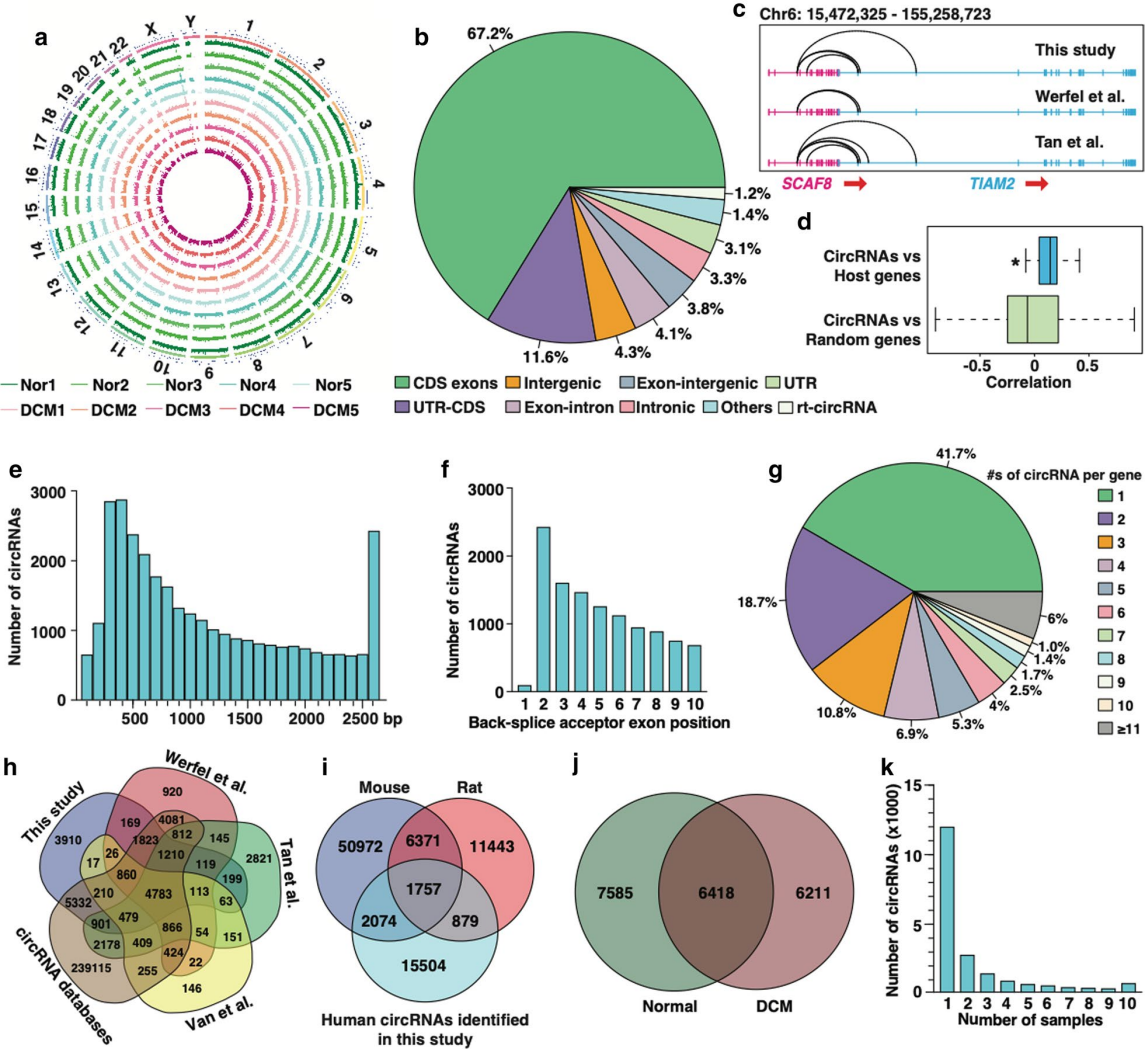
General feature of the circRNAs identified in human heart. **a** The distribution of 20,214 circRNAs identified in human hearts from 5 normal (Nor1-5) and 5 DCM (DCM1-5) patients, was mapped in different chromosomes. The circos plot tracks represent the normalized expression (spliced reads per billion mapping, denoted as SRPBM) of circRNAs in the different samples. **b** Percentage of genomic origins of the identified circRNAs. **c** Schematic diagram showing that rt-circRNAs originate from *SCAF8* (in red) and *TIAM2* (in cyan) genes identified in human heart in this study (upper) and in studies by Werfel et al. (middle) [29] and Tan et al. [27] (bottom). Arrows point to the transcriptional directions of *SCAF8* and *TIAM2* genes. **d** Correlation between the expression of circRNAs and their host genes or randomly chosen linear genes. *p < 0.05. **e** The size distribution of identified circRNAs. **f** Distribution of circRNAs with respect to most upstream circularized exon. **g** Distribution of numbers of circRNAs produced per gene. **h** Four-set venn diagram showing the over-lapping circRNAs identified in human heart in this study and in studies by Tan et al., Werfel et al. and Van et al., as well as 263,738 non-redundant circRNAs obtained by merging human circRNAs in 4 circRNA databases including circBase (n = 91,986), circBank (n = 140,331), circRNADB (n = 32,883), and CIRCpedia V2 (n = 183,943). **i** Venn diagram showing the over-lapping circRNAs identified in human heart in this study and reported mouse and rat circRNAs. The 61,174 non-redundant mouse circRNAs were obtained by merging circRNAs reported by study of Werfel et al. (n = 9474), Jakobi et al. (n = 561), and Tan et al. (n = 2632), as well as 2 circRNA databases including circBase (n = 1756) and CIRCpedia V2 (n = 54,274). The 20,450 non-redundant rat circRNAs were obtained by merging circRNAs reported by study of Werfel et al. (n = 12,256) and CIRCpedia V2 (n = 17,083). **j** Venn diagram showing the number of circRNAs unique or common between normal and DCM human samples. **k** Number of human heart samples in which circRNAs with different abundance were identified

With a threshold cutoff of ≥ 2 unique back-spliced reads present in at least one sample, we identified a total of 20,214 circRNAs in the 10 samples (Fig. 1a and Additional file 5: Table S3). The identified circRNAs originated primarily from the coding-sequence (CDS) regions of exons of single linear genes (67.2%), followed by exons spanning coding and untranslated regions (UTR) (UTR-CDS) (11.6%) (Fig. 1b). Notably, a total of 252 circRNAs (1.2%) were assigned to the exons of two adjacent genes on the same strand (Additional file 6: Table S4), likely representing a novel class of circRNAs termed as rt-circRNA [23]. In particular, the genes *MYH6* and *MYH7* produced the largest number (n = 22) of such circRNAs, which were also observed in human, mouse and rat hearts as reported by two earlier studies (Additional file 7: Figure S3A-C) [27, 29]. In addition, these *MYH6/MYH7*-derived circRNAs were highly abundant and conserved in hearts of human, mouse and rat (Additional file 7: Figure S3D). However, close inspection of the sequence between the exons where they are back-spliced suggests that the back-spliced junctions of these circRNAs are probably artifactual due to read misalignment caused by the high sequence homology between *MYH6* and *MYH7* (Additional file 7: Figure S3E). This result prompted us to employ more stringent criteria to filter circRNAs originating from two neighboring genes with highly homologous sequences. Using this revised filtering strategy, we identified 110 putative rt-circRNAs. Among them, *SCAF8* and *TIAM2* contain the largest number of rt-circRNAs (n = 4), all of which were also previously identified in the human heart (Fig. 1c).

We next examined whether there was a correlative relationship between the expression of circRNAs and their parent linear genes. We found that there was a significantly higher correlation between circRNAs and their linear parent genes compared to that between circRNAs and randomly chosen linear genes (Fig. 1d), suggesting that the generally high-abundance circRNAs tend to have high-abundance linear counterparts. We further found that the length of the majority of identified circRNAs was around 300–400 bp (Fig. 1e). In accordance with previous studies [46–48], the most common splice accepting circularized exon was exon 2 (Fig. 1f). Furthermore, we found that the majority of circRNA-producing genes generated 1 circRNA (41.7%, 2,231/5,350) and only 6.0% (n = 319) produced more than 10 circRNAs (Fig. 1g). The top two genes producing the largest number of circRNAs were *TTN* (Titin) and *RYR2* (Ryanodine receptor 2), which generated 197 and 173 circRNAs, respectively (Additional file 8: Figure S4A-B). Because the *TTN* and *RYR2* genes have the largest number of exons in human genome, we hypothesize that the number of circRNAs produced is positively correlated to the number of exons within their parent genes. Indeed, a proportional increase in the number of circRNAs was observed to correlate with the number of exons per gene (Additional file 8: Figure S4C). Furthermore, we found 17,733 identified circRNAs, in total, contain exons. Among these, single exonic circRNAs were found in 8.4% (1494/17,733) while the rest of circRNAs were encoded by more than two exons (Additional file 8: Figure S4D).

Next we compared the circRNAs that we identified with others that were previously identified in human hearts [27–29] and enlisted in circRNA databases [40, 41, 43, 49]. We found that 16,304 circRNAs (80.7%) were previously reported by either human heart circRNA studies or enlisted in circRNA databases (Fig. 1h). In addition, 3,831 and 2,636 circRNAs have orthologs in the mouse and rat, respectively. Importantly, 1,757 were conserved in all three species by integrating mouse and rat circRNAs reported by previous studies and databases [26, 27, 29, 40, 41, 43, 49] (Fig. 1I). We further found that 7,585 and 6,211 circRNAs were specifically identified in normal and DCM hearts, respectively, and that 6,418 were identified in both (Fig. 1j). Further analysis revealed that only 729 circRNAs were present in all the 10 samples (Fig. 1k), suggesting that many circRNAs exhibit a high degree of variation in expression among human individuals. Additionally, the majority of circRNAs (n = 12,099) were observed in only one sample (Fig. 1k), which likely represent false positives or low-abundance circRNAs. To clarify this, we compared the circRNAs observed once in this study with circRNAs reported by 3 previous human heart circRNA studies [27–29] and 4 circRNA databases [40, 41, 43, 49]. This analysis revealed that 8,666 (71.6%) among them were previously detected (Additional file 9: Figure S5), suggesting these circRNAs observed in a single sample are probably authentic and thus should be retained in studies aiming for circRNA discovery. Collectively, these results indicate that the expression of circRNAs, including rt-circRNAs, is widespread in the human heart with a certain degree of conservation between species.

### Identification of the most abundant circRNAs in healthy human heart

Given the observation that circRNA abundance was not universally distributed across samples (Fig. 1k), we restricted our analysis to 2,461 circRNAs which were considered to be high-confidence circRNAs as they had ≥ 2 reads spanning the back-spliced junction in ≥ 4 samples in at least one group (Additional file 10: Table S5). All these high-confidence circRNAs were overlapped with previously identified circRNAs (Additional file 11: Figure S6A) and 1,121 (45.6%) showed conservation in either mouse or rat (Additional file 11: Figure S6B), indicating the reliability of these circRNAs we identified.

**Fig. 2:**
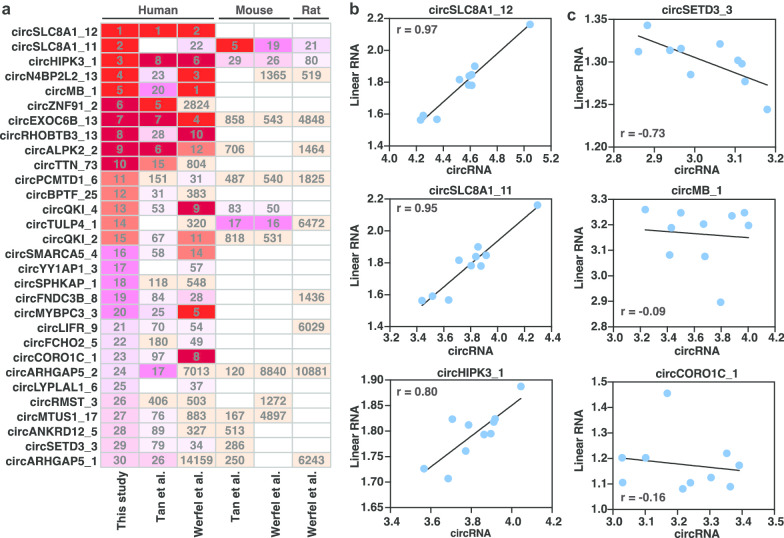
Identification of the most abundant circRNAs in normal human hearts. **a** Heatmap illustrates the ranking of the top 30 most abundant circRNAs identified in normal hearts in this study, and in normal human, mouse and rat hearts that were identified by Tan et al. and Werfel et al. [27, 29]. The numbers in the colored boxes indicate the ranking of the circRNAs within the indicated species and blank boxes represent the circRNA was not detected in the indicated species. **b** Correlation between expression of the top 3 most abundant circRNAs including circSLC8A1_12, circSLC8A1_11 and circHIPK3_1 and their host genes which show positively correlation, as well as **c** circSETD3_3, circMB_1, circCORO1C_1 and their host genes which are independent of each other

CircRNAs that are abundantly expressed in normal heart likely contribute to the maintenance of cardiac homeostasis. We therefore focused on the top 30 circRNAs with the highest average expression level across the 5 healthy heart samples (Fig. 2a). The top 2 most abundant circRNAs (*circSLC8A1_11* and *circSLC8A1_12*) originate from the *SLC8A1* (sodium/calcium exchanger 1) gene. While most of the top circRNAs were also identified in the studies of Tan et al. [27] and Werfel et al. [29], which found high expression levels of circRNAs such as circSLC8A1_12, circHIPK3_1 and circALPK2_2 in healthy hearts, some exceptions were observed. These include circSLC8A1_11 and circYY1AP1_3 that were not detected in study of Tan et al. [27], as well as circZNF91_2 and circPCMTD1_6 whose expression was identified to be abundant in the heart in only one of the previous studies (Fig. 2a). Comparison of the results obtained from different species revealed that half of these highly abundant circRNAs in normal human hearts were conserved in mouse or rat hearts, but only 2 of them including circSLC8A1_11 and circHIPK3_1 exhibited high levels of expression in all species, and 2 of them including circQKI_4 and circTULP4_1 were abundantly expressed in human and mouse hearts, but not in rat. The expression levels of the remaining conserved circRNAs were moderate or low in rodent hearts. We then calculated the correlation between the abundance of top circRNAs and their host genes and found a highly positive correlation for most of these circRNAs (Additional file 12: Figure S7A), including the top 3 most abundant ones (i.e., circSLC8A1_11, circSLC8A1_12 and circHIPK3_1) (Fig. 2b). In contrast, circSETD3_3, circMB_1 and circCORO1C_1, together with some others (Additional file 12: Figure S7B), appeared to be independently transcribed with their linear parent genes (Fig. 2c). Taken together, by integrating results from multiple studies, our analyses define the most abundant circRNAs in the healthy human heart.

### Identification of dysregulated circRNAs in DCM

**Fig. 3:**
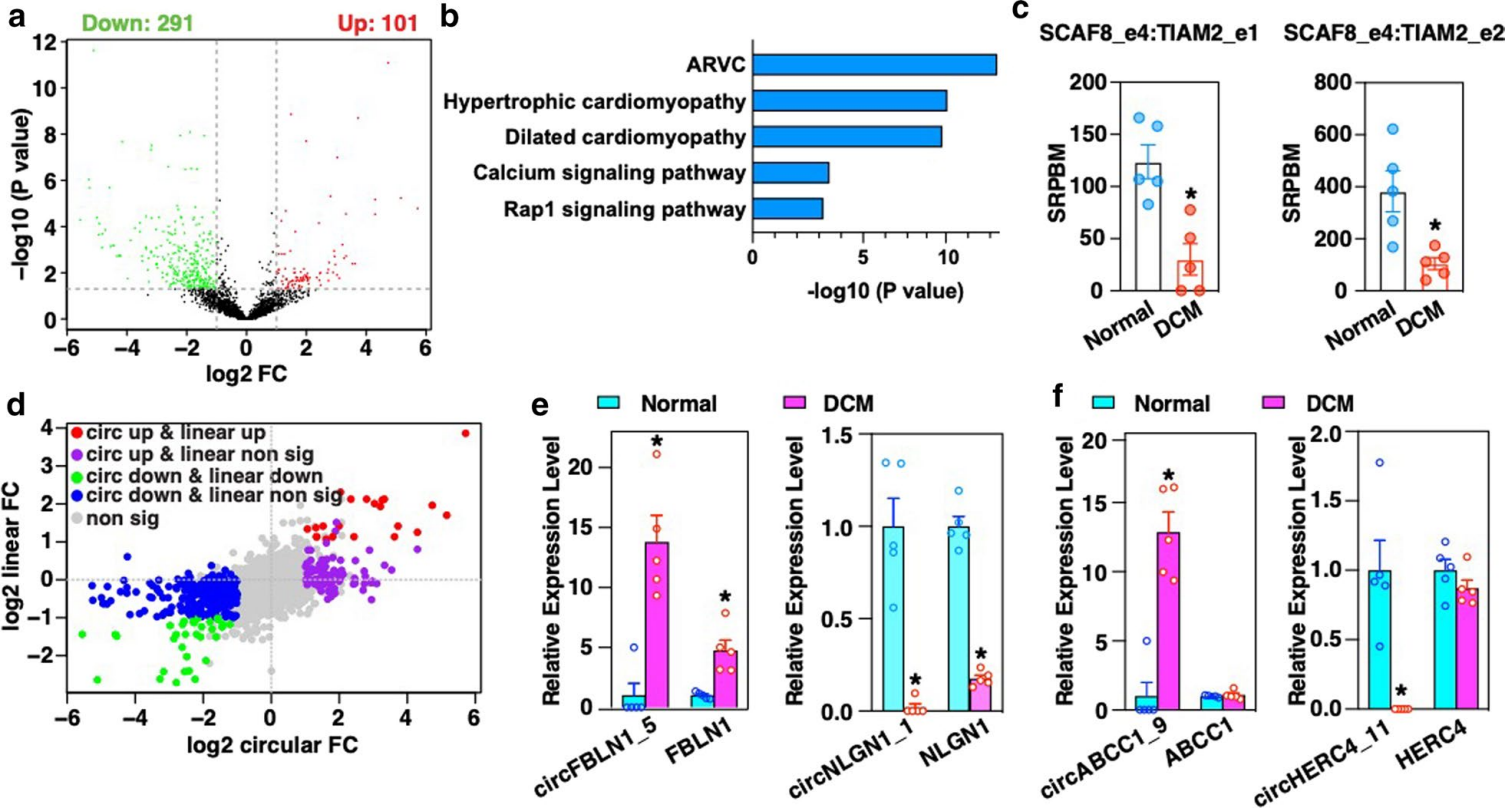
Identification of dysregulated circRNAs in DCM. **a** Volcano plot showing differentially expressed circRNAs in DCM as compared to normal samples (*p* < 0.05 and Fold Change (FC) > 2). **b** Enriched pathway analysis for the host genes of the significantly differentially expressed circRNAs. ARVC: arrhythmogenic right ventricular cardiomyopathy. **c** Down-regulation of 2 rt-circRNAs originated from *SCAF8* and *TIAM2* gene including SCAF8_e4:TIAM2_e1 and SCAF8_e4:TIAM2_e2 in DCM. **d** Correlation of log2 FC of circRNA versus log2 FC of linear RNA expression. **e** Examples of significantly up- and down-regulated circRNAs with expression that is dependent or **f** independent of their host genes. *p < 0.05 (edgeR)

We next performed differential analysis to identify circRNAs that are dysregulated in DCM. Using a threshold of *p* < 0.05 and FC > 2, a total of 392 circRNAs, including 101 up-regulated and 291 down-regulated circRNAs, were identified to be differentially expressed in DCM hearts as compared to normal hearts (Fig. 3a and Additional file 10: Table S5). These differentially expressed circRNAs were derived from 290 unique host genes. Pathway analysis revealed that these host genes are significantly over-represented in pathways associated with diseases of the heart, including arrhythmogenic right ventricular cardiomyopathy (ARVC), hypertrophic cardiomyopathy, and DCM (Fig. 3b). Interestingly, 2 rt-circRNAs originating from *SCAF8* and *TIAM2*, including SCAF8_e4:TIAM2_e1 and SCAF8_e4:TIAM2_e2, were remarkably downregulated in DCM (Fig. 3c). Many of the significantly down- and up-regulated circRNAs were positively correlated with the expression of their host genes such as circFBLN1_5 with *FBLN1* and circNLGN1_1 with *NLGN1*, respectively (Fig. 3d, e). However, the expression of some circRNAs in DCM was independent of changes in their host gene expression (Fig. 3f). Examples of this discordant relationship included circABCC1_9 and circHERC4_11, which was significantly up- and down-regulated in DCM, respectively, while the expression of their host gene mRNAs had no obvious change in DCM (Fig. 3f). Together, these results indicate that dysregulation of circRNAs in DCM tend to originate from heart disease-related gene loci, which include the rt-circRNAs from *SCAF8* and *TIAM2*.

### Screening circRNAs for potential as miRNA sponges

**Fig. 4:**
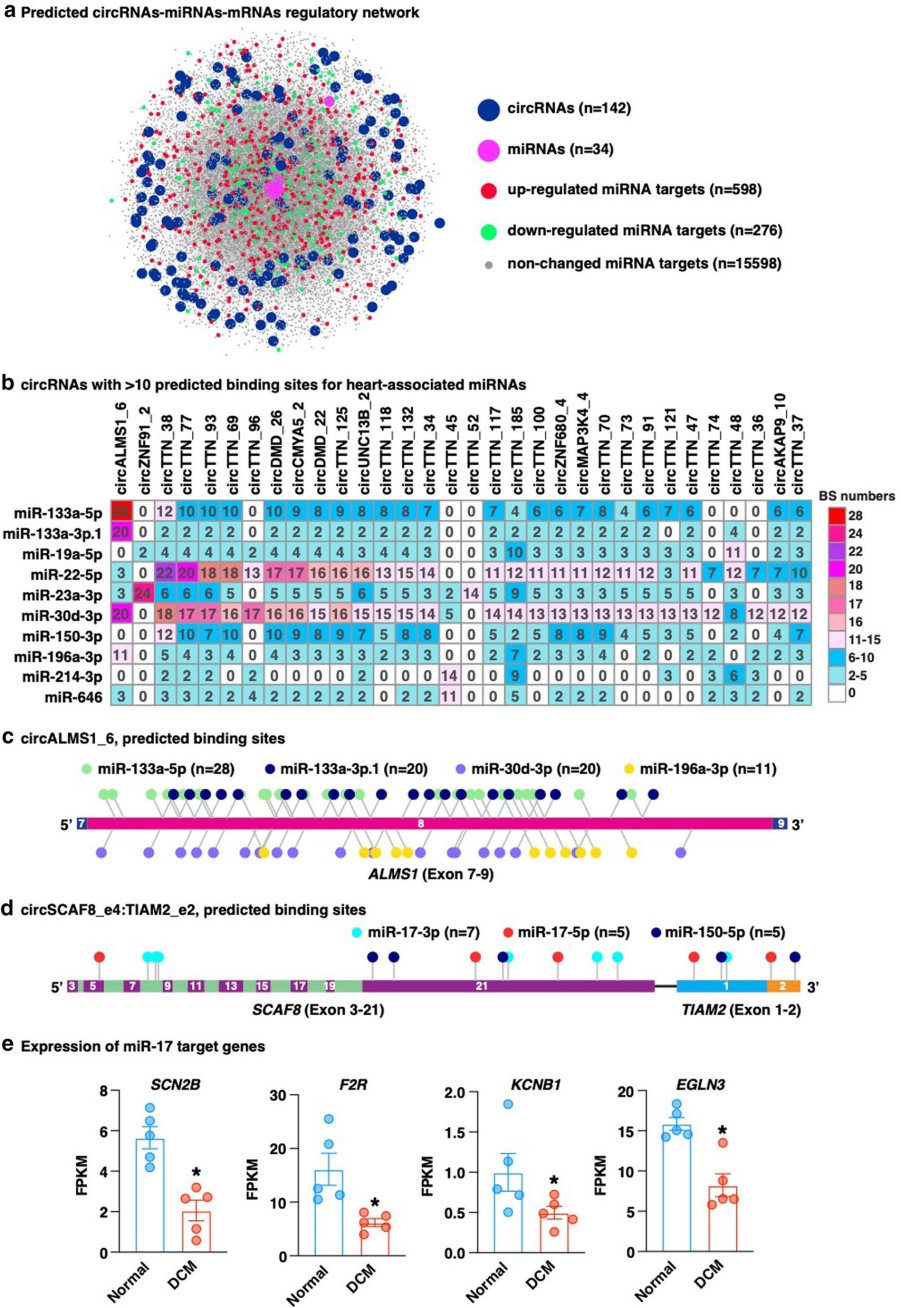
Prediction of circRNA potential for sponging miRNAs. **a** Predicted circRNA-miRNA-circRNA regulatory network. High-abundance circRNAs with at least 5 predicted binding sites (BS) for at least 1 heart disease-related miRNA were included (n = 142). **b** Heatmap showing the number of BS of circRNAs with at least 10 BS for at least one heart disease-related miRNA. **c** Schematic diagram of circALMS1_6 which was predicted to have the strongest potential to sponge miR-133. **d** Schematic diagram of the rt-circRNA, circSCAF8_e4:TIAM2_e2, which was predicted to have potential to sponge multiple heart disease-related miRNAs. Colored rectangles indicate exons and the numbers within them represent the ID of exons. **e** Expression of miR-17 target genes in normal vs DCM human hearts. *p < 0.05 (edgeR)

To screen for cardiac circRNAs with the potential to act as miRNA sponges, we analyzed the sequences of each high-confidence circRNAs for the presence of putative binding sites for 50 miRNAs that have established roles in heart disease (Additional file 2: Table S1). In total, 142 circRNAs were predicted to harbor at least 5 predicted binding sites for at least one of 34 heart disease-related miRNAs (Additional file 13: Table S6). Additionally, a total of 16,472 putative targeted genes of these miRNAs were identified, including 598 and 276 up- and down-regulated genes in DCM hearts compared to normal hearts (Additional file 14: Table S7). A circRNA-miRNA-mRNA regulatory network was further constructed based the predicted interactions between circRNAs and miRNAs, as well miRNAs and mRNAs (Fig. 4a). The top 32 circRNAs that contained more than 10 putative binding sites for at least one miRNA are listed in Fig. 4b. Notably, 23 of these are derived from one gene, *TTN* (Fig. 4b). In particular, circALMS1_6 had the strongest potential to function as miRNA sponge, containing 28 and 20 putative binding sites for miR-133a-5p and miR-133a-3p, respectively (Fig. 4c). Another notable finding is that the rt-circRNA, circSCAF8_e4:TIAM2_e2, was predicted to be capable of sponging multiple miRNAs including miR-150-5p and miR-17 that have been reported to be induced in the rat heart of myocardial ischemia reperfusion injury [50] and in the mouse heart of chronic hypoxia-induced pulmonary hypertension mouse model [51] (Fig. 4d). Interestingly, some of the miR-17 downstream targets, such as *SCN2B* [52, 53], *F2R* [54], *KCNB1* [55] and *EGLN3* [56] that have been implicated in heart dysfunction, were down-regulated in the DCM hearts as revealed by expression analysis of linear RNAs, likely through the unleashed miR-17 that resulted from the down-regulation of circSCAF8_e4:TIAM2_e2 in DCM (Fig. 4e). Collectively, these analyses provide a systematic evaluation of the potential of cardiac circRNAs to sponge miRNAs and provide a resource for studying cardiac miRNA inhibitors.

### Experimental validation of identified circRNAs

**Fig. 5:**
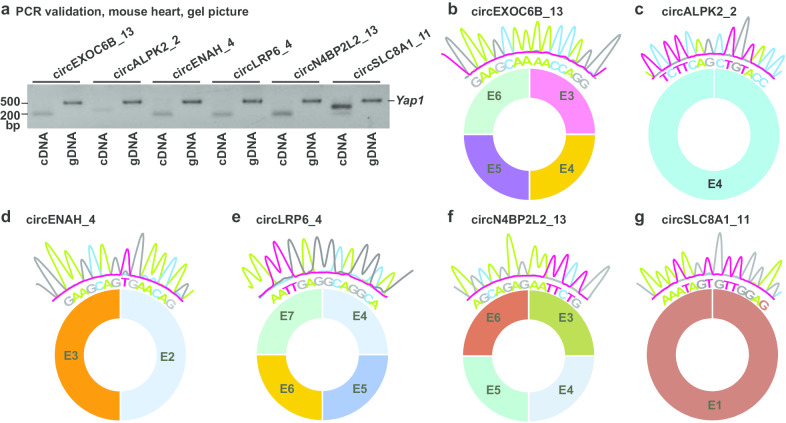
Experimental validation of identified circRNAs. **a** Representative gel pictures showing PCR amplification of the murine orthologs of 6 selected human circRNAs using divergent primers and mouse heart cDNA. Mouse heart genomic DNA served as negative control. Mouse *Yap1* primers served as a positive control for the PCR amplification of genomic DNA. Original uncropped gel is presented in Additional file 15: Figure S8. **b** Sanger sequencing results of the mouse homolog of circEXOC6B_13, **c** circALPK2_2, **d** circENAH_4, **e** circLRP6_4, **f** circN4BP2L2_13 and **g** circSLC8A1_11

Due to lack of availability of human samples, we next sought to validate the conserved circRNAs with mouse orthologs that we identified above using mouse heart samples. We designed divergent primers for the murine orthologs of 6 identified human cardiac circRNAs (Additional file 3: Table S2). These circRNAs were chosen to represent 2 of the most abundant cardiac circRNAs (circEXOC6B_13, circALPK2_2 and circSLC8A_11), 2 of the dysregulated circRNAs (circENAH_4 and circLRP6_4), and 1 non-changed circRNAs between DCM and normal hearts (circN4BP2L2_13). Importantly, these circRNAs have not been experimentally verified by previous studies, with exception of circSLC8A_11 that served as a positive control for circRNA amplification in this study. We performed PCR amplification using mouse heart cDNA as the template, with mouse heart genomic DNA used as a negative control and mouse *Yap1* gene primers used as an internal control for the PCR amplification of genomic DNA. All 6 of the selected circRNAs were successfully amplified in heart cDNA but not in genomic DNA (Fig. 5a). Sanger sequencing of the PCR products verified back-spliced junctions (Fig. 5b–g). Taken together, all the circRNAs examined were confirmed to be bona fide, suggesting the reliability of our in silico circRNA identification.

## Discussion

Herein, we have analyzed rRNA-depleted RNA sequencing datasets from 10 human hearts. By integrating circRNAs that were previously identified in human, mouse and rat hearts [26–29, 40, 41, 43, 49], we have systematically interrogated the global landscape of circRNAs in human heart and have revealed the most abundant circRNAs in healthy hearts, and those that are dysregulated in DCM hearts. Furthermore, we have investigated the potential of human cardiac circRNAs to act as sponges for heart disease-related miRNAs and have generated a list of rt-circRNAs expressed in human heart. Our study adds new information to the previous annotated circRNAs and provides a valuable resource for further functional study of circRNAs in the human heart.

Three earlier studies found that circRNAs are expressed in the human heart and identified a large number of circRNAs [27–29]. The number of circRNAs identified in our study (n = 20,214) is larger than that of previous studies. This could be partially explained by the differences in the genetic background of the human subjects, the algorithms employed and the thresholds used for detecting and filtering circRNAs, and sample size. When comparing the degree of overlap of circRNAs identified in previous studies, congruency was lower than expected despite the use of the same tissue (heart) and species (human). For example, only about half of the circRNAs reported by the three previous studies are in common [27–29] (Fig. 1h). This observation suggests that either the expression pattern of circRNAs varies significantly among different individuals, or that the identified circRNAs in the limited samples were artifactual, which underscores the need to identify a list of reliable cardiac circRNAs by integrating multiple independent studies. Our comparative analysis revealed a total 16,304 cardiac circRNAs that are reproducible in at least one of previous studies (Fig. 1h and Additional file 5: Table S3), which represent a bona fide category of cardiac circRNAs that can be functionally explored in the future.

Our analysis revealed that a significant number of cardiac circRNAs are derived from two different genes, which were also reported by previous studies of circRNAs in heart [27, 29] and brain [16, 36, 46]. Due to the fact that many such circRNAs are derived from neighboring genes with highly homologous sequences, a common practice in circRNA detection is to discard circRNAs from multiple genes [28, 46]. However, a recent breakthrough study showed that a significant portion of circRNAs indeed originate from the exons of two adjacent genes on the same strand. These circRNA are referred to as rt-circRNAs [30] and suggests that circRNAs originated from different genes should not simply be dismissed as false positives, especially in studies with a major focus on circRNA discovery. One striking observation of this study is that a number of circRNAs are identified to be produced from the exons of *MYH6* and *MYH7*, which were also frequently observed, and display high abundance, in hearts of human, mouse and rat by two previous studies (Additional file 7: Figure S3A-D) [27, 29], albeit using different algorithms and cohorts for circRNA detection. This could be particularly interesting if the circRNAs derived from *MYH6* and *MYH7* are bona fide, because these two genes play important roles in healthy and diseased hearts [57]. However, in-depth examination of sequences reveals that these circRNAs are derived from highly homologous region of *MYH6* and *MYH7*, suggesting that these circRNAs are artifacts due to the read misalignment as described by previous studies [28, 46]. Therefore, cautions should be taken when annotating circRNAs from multiple genes. Nevertheless, after filtering out additional circRNAs from the homologous regions of two nearby genes, we identified a total of 110 potentially bona fide rt-circRNAs (Additional file 6: Table S4). Especially, *SCAF8* and *TIAM2* gene produce the largest number of rt-circRNAs (Fig. 1c). Notably, all of the identified putative rt-circRNAs in human heart are not conserved in mouse or rat, suggesting that they are human-specific and recently evolved novel non-coding molecules. Future efforts are needed to validate these rt-circRNAs in greater detail and explore their functional roles in the heart.

Our analysis yielded a list of the top 30 most abundant circRNAs expressed in normal hearts (Fig. 2a). All of the identified top circRNAs, except for circSPHKAP_1 and circRMST_3, are within the top 100 most abundant circRNAs in human or mouse hearts as revealed by at least one previous study (Fig. 2a) [27, 29], suggesting a high degree of fidelity of this top circRNA list. In previous studies, discordant findings have been observed for some of these top circRNAs with regard to their presence and abundance [27, 29]. For instance, circTULP4_1 is absent in the study of Tan et al. [27] but is abundantly expressed in human and mouse heart. Our cross-reference analysis of multiple independent studies helps to reduce confusion over the importance of the identified circRNAs. The function of one of the most abundant circRNAs, circSLC8A1_11, in heart function has been assessed by a recent study [58]. CircHIPK3_1, formed from the circularization of the second exon of *HIPK3*, is another conserved circRNA that is highly expressed in both human and murine heart. An earlier study has shown that circHIPK3_1 plays a critical role in promoting retinal vascular dysfunction in diabetes mellitus [59]. In future studies, it will be interesting to explore the function of circHIPK3_1, as well as some of the other top candidates we have identified such as circTULP4_1 (Fig. 2a), in maintaining cardiac hemostasis. Furthermore, half of the most abundant human circRNAs are conserved in mouse and/or rat. However, only several of these, including circSLC8A1_11, circHIPK3_1, circQKI_4 and circTULP4_1 are observed to be highly expressed in murine heart as well, indicating differences in the circRNA landscape between humans and rodents. In addition, the expression levels of the three human-specific circRNAs including circMB_1, circCORO1C_1 and circSETD3_3 tends to be independent of their host genes, suggesting potentially important roles of these circRNAs in human heart.

Differential analysis revealed that most of the aberrant circRNAs were down-regulated in DCM as compared to normal hearts (Fig. 3a), a feature that is similar to observations in cancer [30, 60]. In cancer, decreased expression of circRNAs was speculated to result from the dilution of circRNA upon cancer cell division [30, 60]. However, this mechanism cannot account for the reduced expression in DCM hearts due to the fact that adult cardiac myocytes have a very limited replicative capacity and extensive cell death leads to a reduction in myocyte number in DCM [61]. Another interesting finding is that many of the abundant circRNAs originate from already-established cardiovascular disease-related gene loci, as indicated by the results of pathway analysis of their host genes (Fig. 3b). For example, four circRNAs generated from the *TTN* locus, including circTTN_34, circTTN_52, circTTN_70 and circTTN_132 were significantly down-regulated in DCM as compared to normal hearts (Additional file 10: Table S5). These data are consistent with an earlier study that reported on the dysregulation of circRNAs synthesized from the *TTN* gene in DCM [62]. The *TTN* gene encodes the protein titin, which is the largest known protein expressed in the heart. The *TTN* gene was also shown to generate the largest number of circRNAs in the heart (Additional file 8: Figure S4A). Another interesting gene which generated two significantly down-regulated circRNAs (circRYR2_71 and circRYR2_95) in DCM, was the *RYR2* gene. RYR2 is the major Ca^2+^ release channel on the sarcoplasmic reticulum and plays a critical role in regulating excitation–contraction coupling and Ca^2+^ homeostasis in the heart [63]. Sequence variations of these two genes that result in altered amino acid sequence and disruption of protein structure and function, have been linked to DCM [64, 65]. There was no evidence to support the presence of mutations in *TTN* and *RYR2* genes in the 5 DCM patients analyzed. Furthermore, RNA-seq analysis revealed that their expression did not change at the mRNA level as compared to controls. Thus the downregulation of circRNAs observed from the *TTN* and *RYR2* genes in DCM likely represent a novel mechanism that awaits further identification. We observed that multiple circRNAs were differentially expressed in DCM, with no obvious change in the parent gene expression (Fig. 3d and f), confirming previous findings that the expression of circRNAs can be independent of their parent genes [66].

One of the best documented functions of circRNAs is to sponge miRNAs, which is similar to linear long non-coding RNAs [67, 68]. Furthermore, the regulatory role of circRNAs via the circRNA-miRNA-mRNA axis has been increasingly implicated in human diseases [69]. In addition, the relatively higher stability of circRNAs compared to linear mRNAs due to the lack of free 5′ and 3′ ends allow them to be ideal molecular sponges for miRNAs. Our results in predicting the ability of circRNAs to bind heart disease-related miRNAs provides a guideline for further exploration of the regulatory roles of cardiac circRNAs (Fig. 4a, Additional file 13: Table S6, and Additional file 14: Table S7). Notably, circRNAs produced from the *TTN* gene show a strong potential to bind miRNAs, suggesting that the function of the cardiac *TTN* gene may have added complexity. In addition, circALMS1_6 has strong potential to sponge miR-133, a well-known miRNA that is abundantly expressed in skeletal muscle and cardiomyocytes [70]. Given the important roles of miR-133 in regulating cardiac hypertrophy, it will be interesting to determine whether circALMS1_6 affects cardiac hemostasis via inhibition of miRNAs. Furthermore, we identified a rt-circRNA, circSCAF8_e4:TIAM2_e2, with the potential to bind multiple miRNAs including miR-17 that have been shown to be induced in diseased hearts [50, 51] (Fig. 4d). In DCM hearts, the expression of circSCAF8_e4:TIAM2_e2 is decreased (Fig. 3c). It is therefore possible that the down-regulation of this rt-circRNA contributes to the up-regulation of miR-17 via releasing the sequestration of miR-17 interacted with, thereby inhibiting the down-stream targets of miR-17. In support of this hypothesis, several miR-17 targets with known cardiac function are down-regulated in DCM hearts (Fig. 4e). One interesting target is *SCN2B* that encodes voltage-gated sodium channel β2-subunits. The sequence variations of *SCN2B* have been associated with human cardiac arrhythmias [53] and genetic deletion of *Scn2b* in mice leads to ventricular and atrial arrhythmias [52]. Our study suggests a possible regulatory role of circSCAF8_e4:TIAM2_e2-miR-17-*SCN2B* axis in heart and expands our understanding of the function of the newly discovered rt-circRNAs.

## Conclusions

In conclusion, our study provides a list of putative key circRNAs that are expressed in normal and DCM human hearts. These circRNAs we identified are helpful to guide future functional studies. Given their abundance and multiple functional roles, circRNAs, including rt-circRNAs, are expected to be a group of emerging players in the regulation of human heart diseases.

## Supplementary information

**Additional file 1**. ** Figure S1**. Flowchart for de novo identification of circRNAs in human heart.

**Additional file 2**. ** Table S1**. Information of DCM-related miRNAs used for analysis of sponge potential. The seed sequence of each miRNA was obtained from TargetScan database. PMID is the PubMed number for studies which reported the miRNAs.

**Additional file 3**. ** Table S2**. Primers used for validation of the mouse ortholog of selected human circRNAs. Mouse ***Yap1*** primers were used as internal control for PCR reaction using genomic DNA.

**Additional file 4**. ** Figure S2**. Validation of the public RNA-seq dataset. (A) Principal Component Analysis (PCA) of the 10 samples (5 normal and 5 DCM hearts) using expression of genes with raw count > 10 in at least 5 samples. (B) Expression of ***NPPA*** in normal and DCM hearts from the RNA-seq dataset. PFKM: Fragments Per Kilobase of exon per Million fragments mapped. *p < 0.05 (edgeR).

**Additional file 5**. **Table S3**. Information of the 20,214 circRNAs identified in this study. Chromosome coordinates are in hg38 annotation.

**Additional file 6**. ** Table S4**. List of circRNAs originated from two different genes in 10 human heart samples. Chromosome coordinates are in hg38 annotation. In the last column, “yes” indicates that the circRNA is from two neighboring genes with highly similar sequence, and “no” indicates no sequence similarity was observed and the circRNA is likely a bona fide rt-circRNA.

**Additional file 7**. ** Figure S3**. Analysis of artifactual circRNAs from MYH6 and MYH7 gene. (A) Schematic diagram showing a number of rt-circRNAs originate from ***MYH6*** and ***MYH7*** genes in human heart identified in this study, study of Tan et al. [27] and Werfel et al. [29], and in (B) mouse heart identified in this study and study of Werfel et al. [29], as well as in (C) rat heart revealed in the study of Werfel et al. [29]. Arrows point to the transcriptional directions of ***MYH6*** and ***MYH7*** genes. (D) Heatmap illustrates the rank of the average expression across all studied samples for circRNAs produced from ***MYH6*** and ***MYH7*** genes in this study, and in human, mouse and rat hearts as identified by Tan et al. [27] and Werfel et al. [29]. The numbers in the colored boxes indicate the rank of the circRNAs within the indicated species. (E) Representative example of circMYH6_e35:MYH7_e37 that is likely artifact. The upper schematic diagram illustrating the formation of circMYH6_e35:MYH7_e37 due to read misalignment. The exon 35 of ***MYH6*** (red, the donor exon) and exon 36 of ***MYH7*** (yellow, upstream of the acceptor exon) are homologous. The exon 36 of ***MYH6*** (green, downstream of the donor exon) and exon 37 of ***MYH7*** (blue, the acceptor exon) are highly homologous. Considering a chimeric sequencing read that is supposedly to be aligned back to exon 35 (red line without arrow) and 36 (green line with arrow) of ***MYH6***, due to the high sequence similarity, the down-stream part of the read (green line with arrow) may be misaligned to exon 37 of ***MYH7*** (blue line with arrow), leading to the artifactual identification of back-spliced junction, and versa vice. Dashed lines indicate splicing junctions. The bottom panel shows the sequence alignment of linear MYH6_e35:MYH6_e36, MYH7_e36:MYH7_e37, and the predicted sequence of the circular MYH6_e35:MYH7_e37.

**Additional file 8**.** Figure S4**. Relationship between numbers of circRNAs and exons of their host genes. (A) Schematic diagram of all the circRNAs originated from ***TTN*** and (B) ***RYR2*** gene. Arrows point to the transcriptional directions of ***TTN*** and ***RYR2*** genes. (C) The numbers of circRNAs increased proportionally with the numbers of exons per gene (binned to 10). (D) Distribution of exon numbers to produce circRNAs.

**Additional file 9**. ** Figure S5**. Comparison of circRNAs with 2 unique back-spliced reads observed only once in this study with human circRNAs reported by 3 previous studies and 4 circRNA databases. (A) Four-set venn diagram showing the number of over-lapping circRNAs between the 12,099 circRNAs with 2 unique back-spliced reads observed only in one sample in this study, and human cardiac circRNAs reported by 3 previous studies. (B) Five-set venn diagram showing the number of over-lapping circRNAs between the 12,099 circRNAs with 2 unique back-spliced reads observed only in one sample in this study, and human circRNAs reported by 4 circRNA databases. (C) Three-set venn diagram showing the number of over-lapping circRNAs between the 12,099 circRNAs with 2 unique back-spliced reads observed only in one sample in this study, and 24,256 and 263,738 non-redundant circRNAs obtained by merging circRNAs reported by study of Werfel et al. (n = 16,427), Tan et al. (n = 15,303), and Van et al. (n = 8878), as well as 4 circRNA datasets including circBase (n = 91,986), circBank (n = 140,331), circRNADB (n = 32,883), and CIRCpedia (n = 183,943).

**Additional file 10**. **Table S5**. List of high-confidence and significantly dysregulated circRNAs in DCM compared to normal heart samples. Chromosome coordinates are in hg38 annotation. The threshold for significance was* p* <0.05 and fold change > 2.

**Additional file 11**.** Figure S6**. Comparison of the 2461 high-abundance circRNAs with reported human, mouse and rat circRNAs. (A) Three-set venn diagram showing the number of over-lapping circRNAs between the 2461 high-abundance circRNAs identified in this study, and 24,256 and 263,738 non-redundant circRNAs obtained by merging circRNAs reported by study of Werfel et al. (n = 16,427), Tan et al. (n = 15,303), and Van et al. (n = 8,878), as well as 4 circRNA datasets including circBase (n = 91,986), circBank (n = 140,331), circRNADB (n = 32,883), and CIRCpedia (n = 183,943). (B) Three-set venn diagram showing the number of over-lapping circRNAs between the 2461 high-abundance circRNAs identified in this study, and 61,174 and 20,450 reported mouse and rat circRNAs. The 61,174 non-redundant mouse circRNAs were obtained by merging circRNAs reported by study of Werfel et al. (n = 9474), Jakobi et al. (n = 561), and Tan et al. (n = 2632), as well as 2 circRNA databases including circBase (n = 1756) and CIRCpedia V2 (n = 54,274). The 20,450 non-redundant rat circRNAs were obtained by merging circRNAs reported by study of Werfel et al. (n = 12,256) and CIRCpedia V2 (n = 17,083).

**Additional file 12**. ** Figure S7**. Correlation of expression between 24 the most abundant circRNAs and their host genes. (A) CircRNAs with expression significantly correlated with the expression of host genes (Pearson correlation,* p* < 0.05). (B) CircRNAs with expression independent of their host genes (Pearson correlation,* p* > 0.05).

**Additional file 13**. ** Table S6**. CircRNAs with potential to sponge heart disease-related miRNAs. CircRNAs with at least 5 predicted binding sites for at least one miRNA were listed.

**Additional file 14**. **Table S7**. Predicted targets of heart disease-related miRNAs. The targeted genes of each miRNA were obtained from TargetScan (http://www.targetscan.org/vert_72/). The significance of changes of targeted genes in DCM compared to normal samples were determined by DESeq2 with threshold of fold change >2 and FDR <0.05. A total of 937 and 480 genes were identified to be significantly up- and down-regulated in DCM, respectively.

**Additional file 15**. ** Figure S8**. Original uncropped PCR gel picture (related to Fig. 5a)

## Data Availability

The RNA-seq data used in this study was downloaded from NCBI database under Accession Number SRP108571.

## Abbreviations

circRNA: Circular RNA
rt-circRNA: Read-through circular RNA
HF: Heart failure
DCM: Dilated cardiomyopathy
PCA: Principle component analysis
UTR: Untranslated region
CDS: Coding sequence
FC: Fold change
ARVC: Arrhythmogenic right ventricular cardiomyopathy
FPKM: Fragments per kilobase of exon per million fragments mapped
SRPBM: Spliced reads per billion mapped reads
