# Supplementary figures and images for: Genomic analysis of circular RNAs in heart
Source: BMC Med Genomics. 2020 Nov 7;13:167. doi: 10.1186/s12920-020-00817-7 (PMC7648966; doi:10.1186/s12920-020-00817-7)

**Supplemental Figure S1**

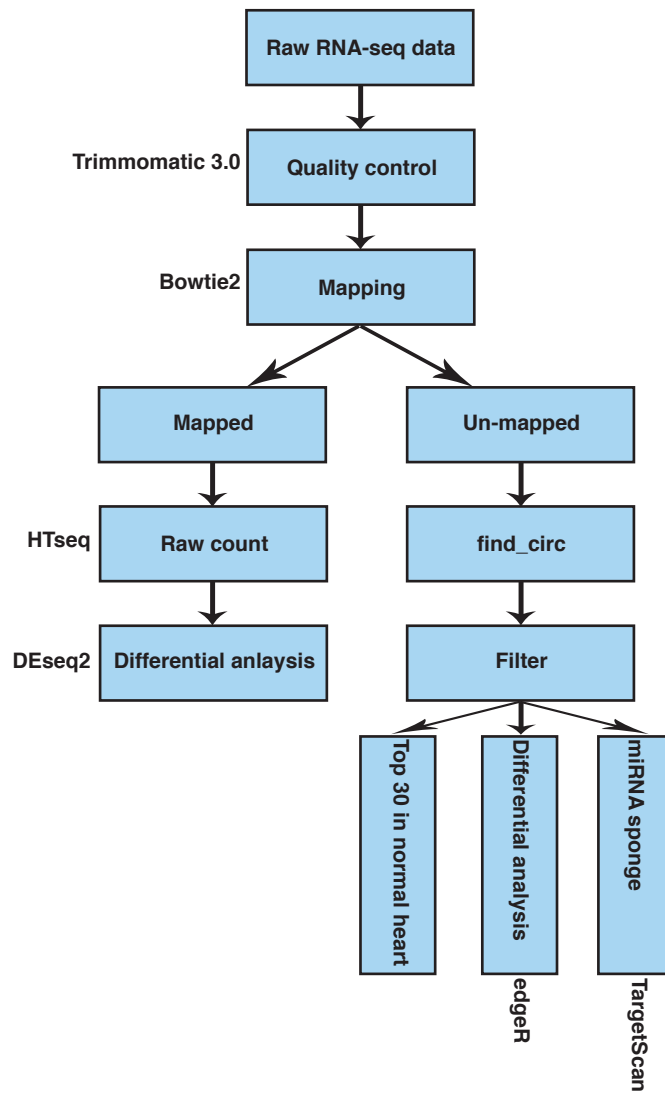

Supplement: Supplementary file 1 — Additional file 1. Figure S1. Flowchart for de novo identification of circRNAs in human heart. [file 12920_2020_817_MOESM1_ESM.pdf]

Supplemental Figure S2

A

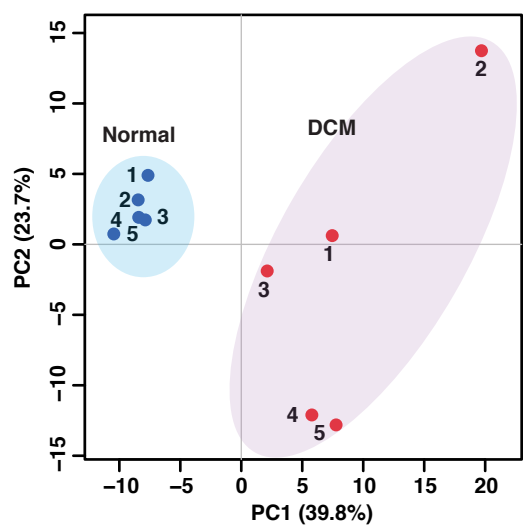

B

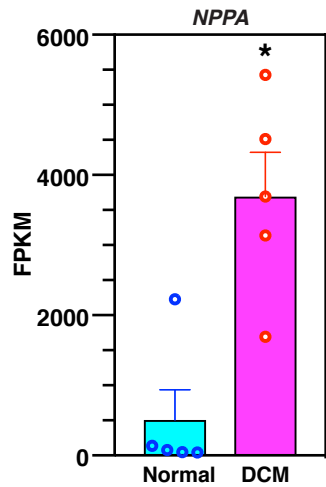

Supplement: Supplementary file 4 — Additional file 4. Figure S2. Validation of the public RNA-seq dataset. (A) Principal Component Analysis (PCA) of the 10 samples (5 normal and 5 DCM hearts) using expression of genes with raw count > 10 in at least 5 samples. (B) Expression of NPPA in normal and DCM hearts from the RNA-seq dataset. PFKM: Fragments Per Kilobase of exon per Million fragments mapped. *p < 0.05 (edgeR). [file 12920_2020_817_MOESM4_ESM.pdf]

Supplemental Figure S4

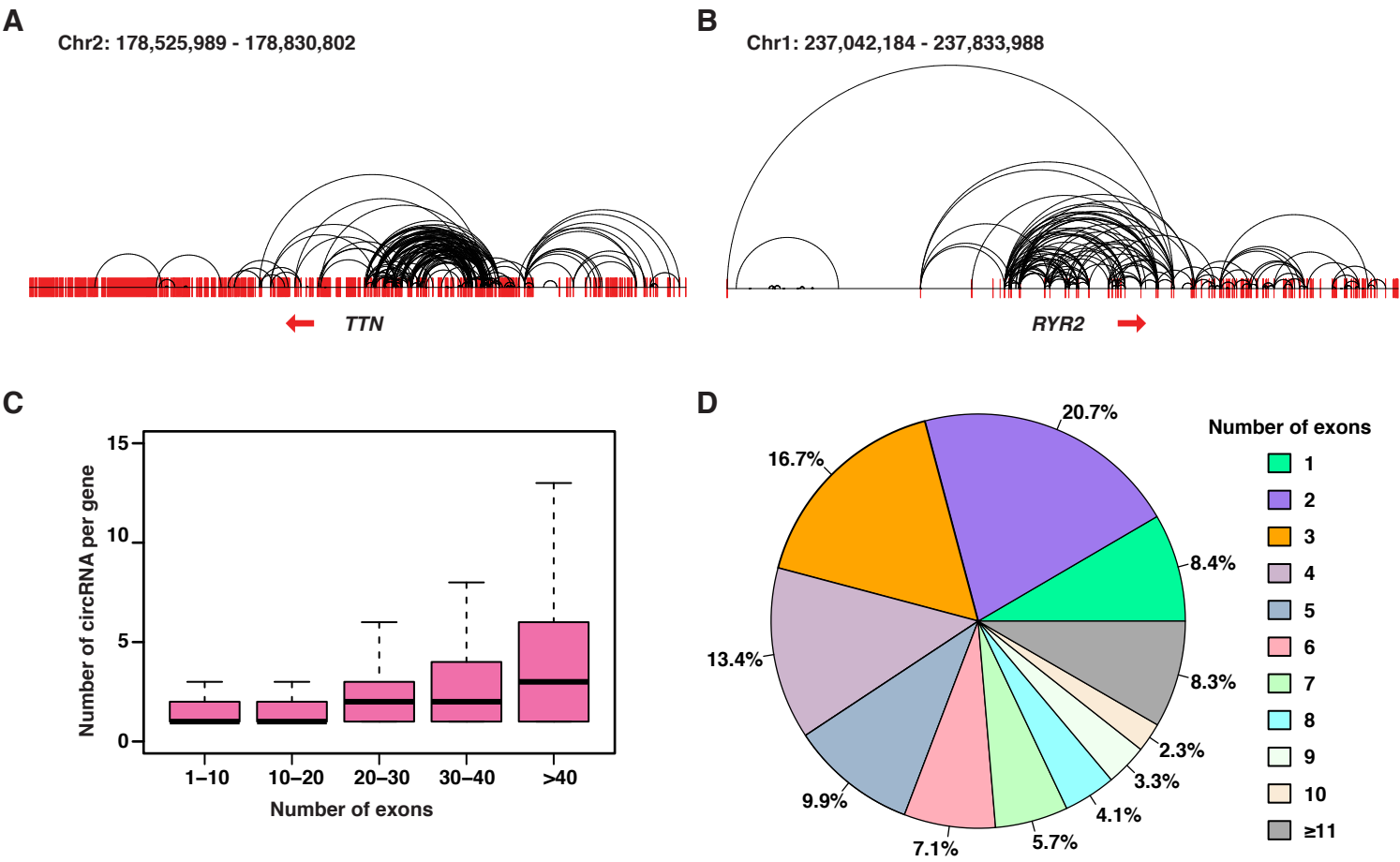

Supplement: Supplementary file 8 — Additional file 8. Figure S4. Relationship between numbers of circRNAs and exons of their host genes. (A) Schematic diagram of all the circRNAs originated from TTN and (B) RYR2 gene. Arrows point to the transcriptional directions of TTN and RYR2 genes. (C) The numbers of circRNAs increased proportionally with the numbers of exons per gene (binned to 10). (D) Distribution of exon numbers to produce circRNAs. [file 12920_2020_817_MOESM8_ESM.pdf]

Supplemental Figure S6

A

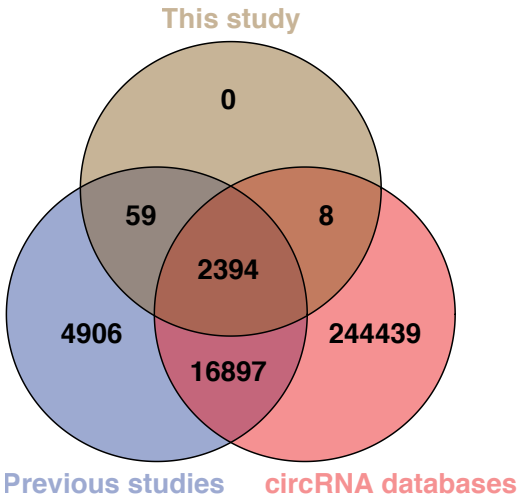

B

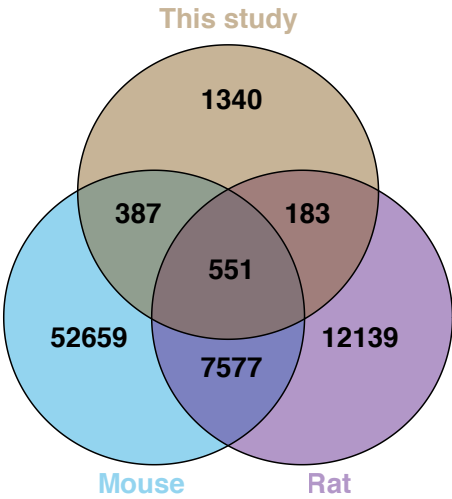

Supplement: Supplementary file 11 — Additional file 11. Figure S6. Comparison of the 2461 high-abundance circRNAs with reported human, mouse and rat circRNAs. (A) Three-set venn diagram showing the number of over-lapping circRNAs between the 2461 high-abundance circRNAs identified in this study, and 24,256 and 263,738 non-redundant circRNAs obtained by merging circRNAs reported by study of Werfel et al. (n = 16,427), Tan et al. (n = 15,303), and Van et al. (n = 8,878), as well as 4 circRNA datasets including circBase (n = 91,986), circBank (n = 140,331), circRNADB (n = 32,883), and CIRCpedia (n = 183,943). (B) Three-set venn diagram showing the number of over-lapping circRNAs between the 2461 high-abundance circRNAs identified in this study, and 61,174 and 20,450 reported mouse and rat circRNAs. The 61,174 non-redundant mouse circRNAs were obtained by merging circRNAs reported by study of Werfel et al. (n = 9474), Jakobi et al. (n = 561), and Tan et al. (n = 2632), as well as 2 circRNA databases including circBase (n = 1756) and CIRCpedia V2 (n = 54,274). The 20,450 non-redundant rat circRNAs were obtained by merging circRNAs reported by study of Werfel et al. (n = 12,256) and CIRCpedia V2 (n = 17,083). [file 12920_2020_817_MOESM11_ESM.pdf]

Supplemental Figure S7

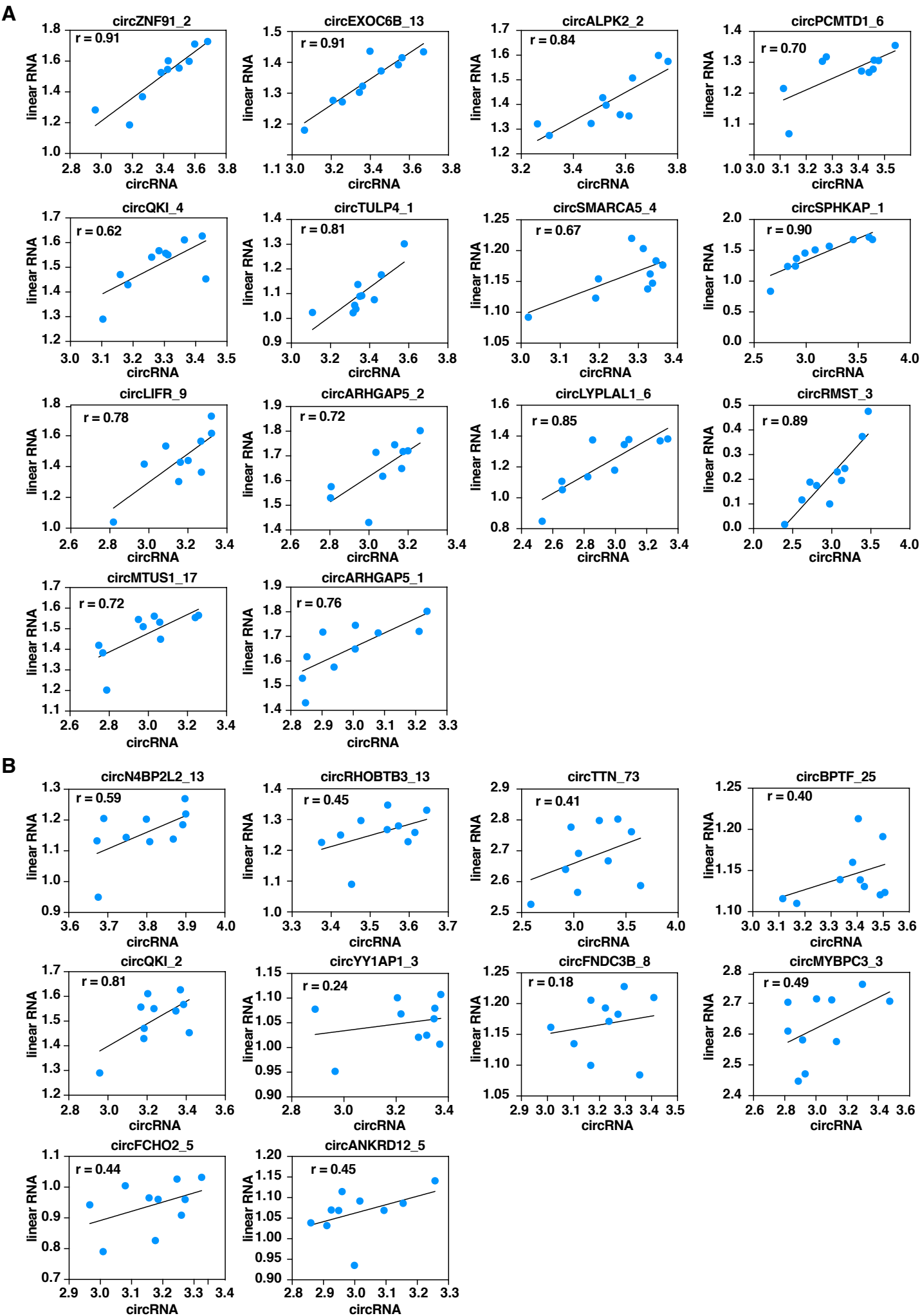

Supplement: Supplementary file 12 — Additional file 12. Figure S7. Correlation of expression between 24 the most abundant circRNAs and their host genes. (A) CircRNAs with expression significantly correlated with the expression of host genes (Pearson correlation, p < 0.05). (B) CircRNAs with expression independent of their host genes (Pearson correlation, p > 0.05). [file 12920_2020_817_MOESM12_ESM.pdf]
