# Supplementary material for: Genomic analysis of circular RNAs in heart
Source: BMC Med Genomics. 2020 Nov 7;13:167. doi: 10.1186/s12920-020-00817-7 (PMC7648966; doi:10.1186/s12920-020-00817-7)

Supplemental Figure S3

A Human, hg38, Chr14: 23,381,990 - 23,435,718

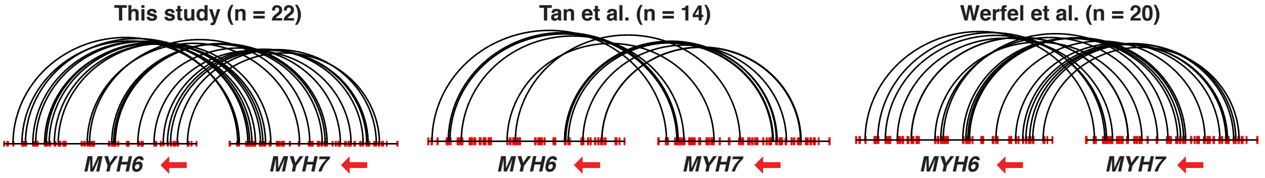

B Mouse, mm10, Chr14: 54,941,921 - 54,994,626

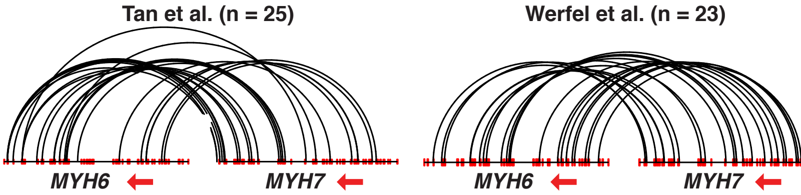

C Rat, rn5, Chr15: 37,492,599 - 37,542,688

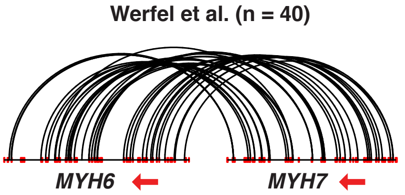

D Rank of circRNAs arising from *MYH6* and *MYH7* based on the abundance

circRNA	H: This study	H: Tan et al.	H: Werfel et al.	M: Tan et al.	M: Werfel et al.	R: Werfel et al.
circMYH6_e32:MYH7_e34	1	8		5		3
circMYH6_e14:MYH7_e16	2			6		7
circMYH6_e29:MYH7_e31	3			12		
circMYH6_e33:MYH7_e35	4					2437
circMYH6_e18:MYH7_e20	5	5				
circMYH6_e35:MYH7_e37	7	29	4	8		
circMYH6_e19:MYH7_e21	8	54				4
circMYH6_e34:MYH7_e36	10	59			115	14
circMYH6_e30:MYH7_e32	11				29	9
circMYH6_e28:MYH7_e30	12	25		1766		12
circMYH6_e37:MYH7_e39	13	46		9	9	10
circMYH6_e17:MYH7_e19	14	7		1		8
circMYH6_e23:MYH7_e25	15	14		3		5
circMYH6_e36:MYH7_e38	19			8508		
circMYH6_e12:MYH7_e13	31					4915
circMYH6_e4:MYH7_e5	45	66		2		1
circMYH6_e7:MYH7_e8	48	30				
circMYH6_e22:MYH7_e24	60	193	5	44		21
circMYH6_e31:MYH7_e33	540					
circMYH6_e3:MYH7_e4	1298	5747		2416		2
circMYH6:MYH7_2	1556	160				6488
circMYH6:MYH7_1	4310			9431		6786

Rank: 1-5; 6-10; 11-20; 21-30; 31-100; >100; ND

E Diagram of circMYH6_35:MYH7_e37 formation

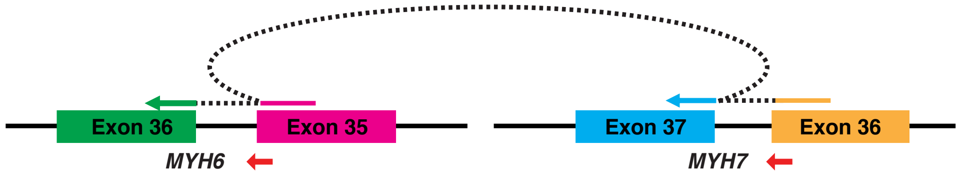

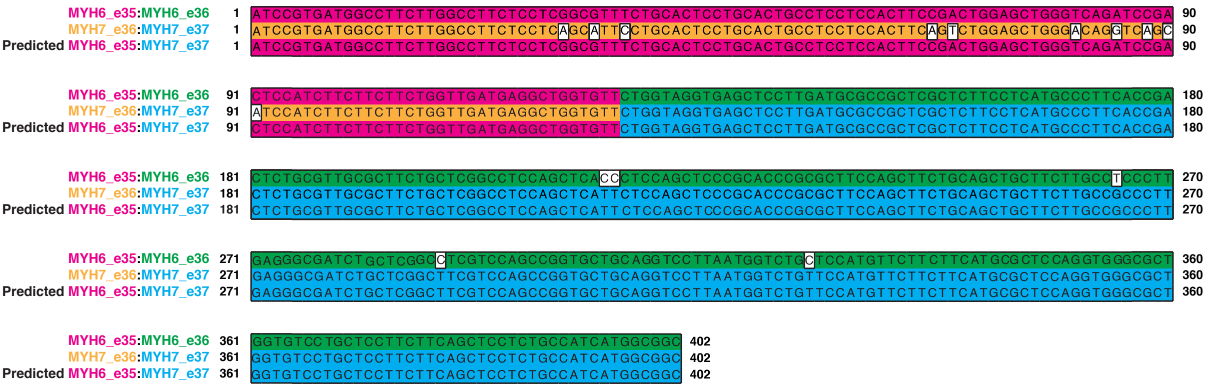

Supplement: Supplementary file 7 — Additional file 7. Figure S3. Analysis of artifactual circRNAs from MYH6 and MYH7 gene. (A) Schematic diagram showing a number of rt-circRNAs originate from MYH6 and MYH7 genes in human heart identified in this study, study of Tan et al. [27] and Werfel et al. [29], and in (B) mouse heart identified in this study and study of Werfel et al. [29], as well as in (C) rat heart revealed in the study of Werfel et al. [29]. Arrows point to the transcriptional directions of MYH6 and MYH7 genes. (D) Heatmap illustrates the rank of the average expression across all studied samples for circRNAs produced from MYH6 and MYH7 genes in this study, and in human, mouse and rat hearts as identified by Tan et al. [27] and Werfel et al. [29]. The numbers in the colored boxes indicate the rank of the circRNAs within the indicated species. (E) Representative example of circMYH6_e35:MYH7_e37 that is likely artifact. The upper schematic diagram illustrating the formation of circMYH6_e35:MYH7_e37 due to read misalignment. The exon 35 of MYH6 (red, the donor exon) and exon 36 of MYH7 (yellow, upstream of the acceptor exon) are homologous. The exon 36 of MYH6 (green, downstream of the donor exon) and exon 37 of MYH7 (blue, the acceptor exon) are highly homologous. Considering a chimeric sequencing read that is supposedly to be aligned back to exon 35 (red line without arrow) and 36 (green line with arrow) of MYH6, due to the high sequence similarity, the down-stream part of the read (green line with arrow) may be misaligned to exon 37 of MYH7 (blue line with arrow), leading to the artifactual identification of back-spliced junction, and versa vice. Dashed lines indicate splicing junctions. The bottom panel shows the sequence alignment of linear MYH6_e35:MYH6_e36, MYH7_e36:MYH7_e37, and the predicted sequence of the circular MYH6_e35:MYH7_e37. [file 12920_2020_817_MOESM7_ESM.pdf]
