# Supplementary material for: Genomic analysis of circular RNAs in heart
Source: BMC Med Genomics. 2020 Nov 7;13:167. doi: 10.1186/s12920-020-00817-7 (PMC7648966; doi:10.1186/s12920-020-00817-7)

Supplemental Figure S5

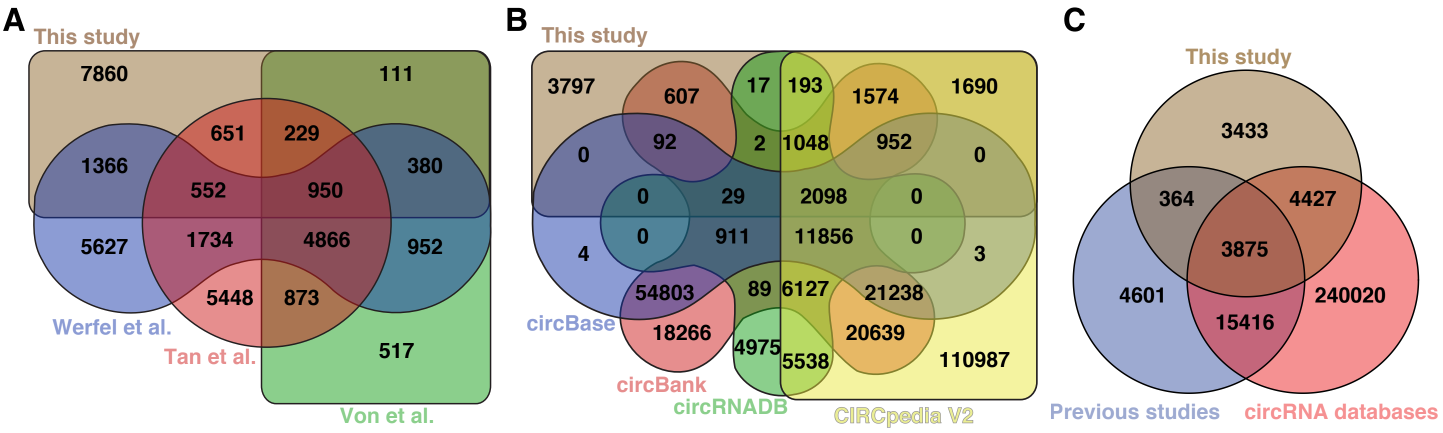

Supplement: Supplementary file 9 — Additional file 9. Figure S5. Comparison of circRNAs with 2 unique back-spliced reads observed only once in this study with human circRNAs reported by 3 previous studies and 4 circRNA databases. (A) Four-set venn diagram showing the number of over-lapping circRNAs between the 12,099 circRNAs with 2 unique back-spliced reads observed only in one sample in this study, and human cardiac circRNAs reported by 3 previous studies. (B) Five-set venn diagram showing the number of over-lapping circRNAs between the 12,099 circRNAs with 2 unique back-spliced reads observed only in one sample in this study, and human circRNAs reported by 4 circRNA databases. (C) Three-set venn diagram showing the number of over-lapping circRNAs between the 12,099 circRNAs with 2 unique back-spliced reads observed only in one sample in this study, and 24,256 and 263,738 non-redundant circRNAs obtained by merging circRNAs reported by study of Werfel et al. (n = 16,427), Tan et al. (n = 15,303), and Van et al. (n = 8878), as well as 4 circRNA datasets including circBase (n = 91,986), circBank (n = 140,331), circRNADB (n = 32,883), and CIRCpedia (n = 183,943). [file 12920_2020_817_MOESM9_ESM.pdf]
