# Supplementary material for: Genomic analysis of circular RNAs in heart
Source: BMC Med Genomics. 2020 Nov 7;13:167. doi: 10.1186/s12920-020-00817-7 (PMC7648966; doi:10.1186/s12920-020-00817-7)

Additional file 15: uncropped gel picture (related to Figure 5A)

Representative gel pictures showing PCR amplification of the murine orthologs of 6 selected human circRNAs using divergent primers and mouse heart cDNA. Mouse heart genomic DNA served as negative control. Mouse *Yap1* primers served as a positive control for the PCR amplification of genomic DNA.

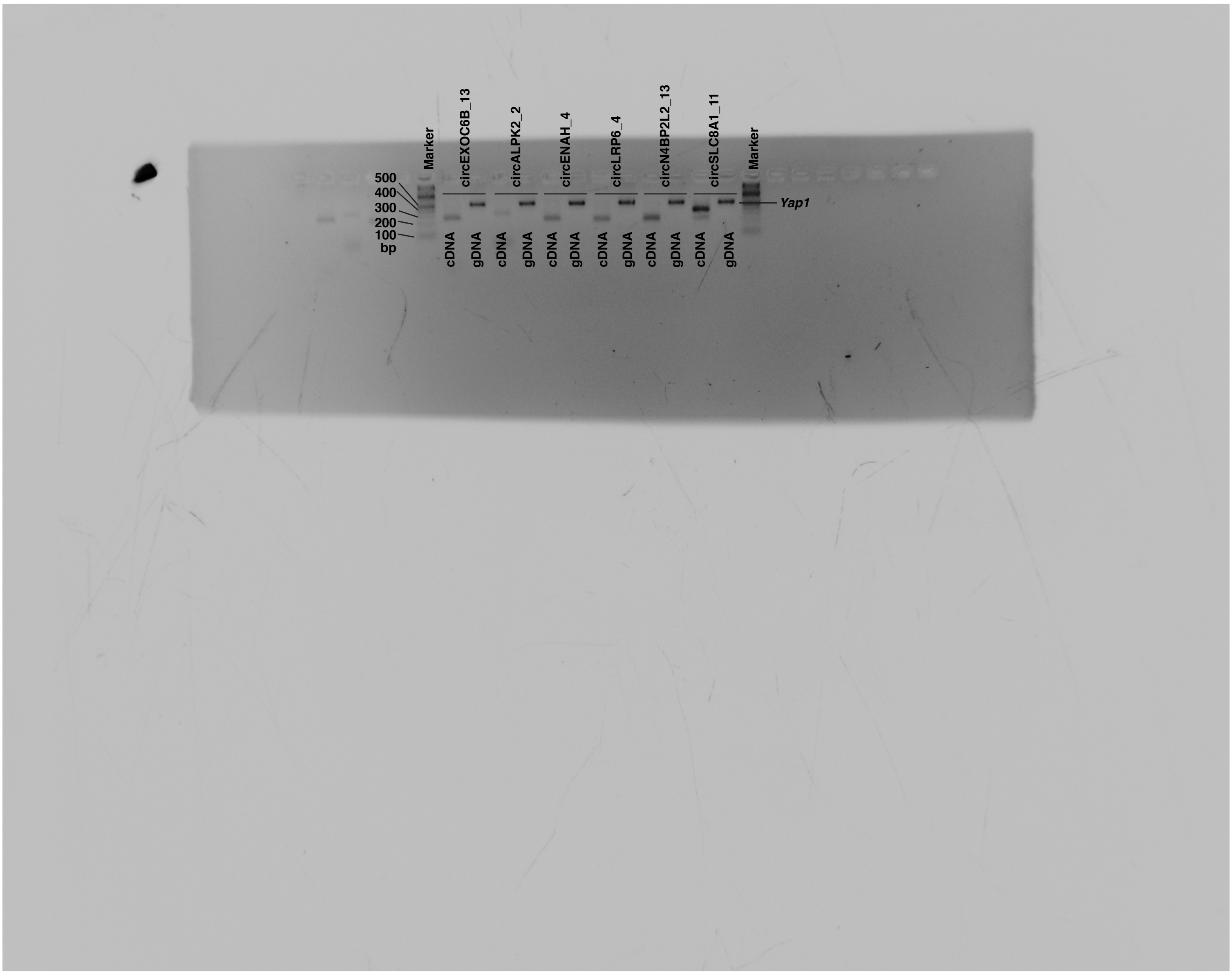

Supplement: Supplementary file 15 — Additional file 15. Figure S8. Original uncropped PCR gel picture (related to Fig. 5a) [file 12920_2020_817_MOESM15_ESM.pdf]
